# Rapid Microbiome Changes in Freshly Deposited Cow Feces under Field Conditions

**DOI:** 10.3389/fmicb.2016.00500

**Published:** 2016-04-13

**Authors:** Kelvin Wong, Timothy I. Shaw, Adelumola Oladeinde, Travis C. Glenn, Brian Oakley, Marirosa Molina

**Affiliations:** ^1^Ecosystems Research Division, United States Environmental Protection Agency, AthensGA, USA; ^2^Oak Ridge Institute for Science and Education, Oak RidgeTN, USA; ^3^Institute of Bioinformatics, University of Georgia, AthensGA, USA; ^4^Department of Computational Biology, St Jude Children’s Research Hospital, MemphisTN, USA; ^5^Ecosystems Research Division, United States Environmental Protection Agency, AthensGA, USA; ^6^Department of Environmental Health Science, University of Georgia, AthensGA, USA; ^7^College of Veterinary Medicine, Western University of Health Sciences, PomonaCA, USA

**Keywords:** metagenomics, cattle feces, microbiome changes, oxygen exposure, sunlight exposure, fecal contamination

## Abstract

Although development of next generation sequencing (NGS) has substantially improved our understanding of the microbial ecology of animal feces, previous studies have mostly focused on freshly excreted feces. There is still limited understanding of the aging process dynamics of fecal microbiomes in intact cowpats exposed to natural environments. Fresh cowpats were sampled at multiple time points for 57 days under field conditions; half the samples were exposed to sunlight (unshaded) while the other half was protected from sunlight (shaded). The 16SRNA hypervariable region 4 was amplified from each sample and sequenced on an Illumina MiSeq Platform. While *Clostridia, Bacteroidia*, and *Sphingobacteria* were dominant classes of bacteria in fresh cowpats, *Alphaproteobacteria, Betaproteobacteria, Actinobacteria*, and *Bacilli* were the dominant classes by the end of the study, indicating a general shift from anaerobic to aerobic bacterial populations. This change was most likely influenced by the shift from cattle gut (anaerobic) to pasture ground (aerobic). Reduced moisture in cowpats may also contribute to the community shift since air can penetrate the dryer cowpat more easily. Twelve genera consisting pathogenic bacteria were detected, with *Mycobacterium, Bacillus*, and *Clostridium* being the most abundant; their combined abundance accounts for 90% of the total pathogenic genera. Taxonomic richness and diversity increased throughout the study for most samples, which could be due to bacteria regrowth and colonization of bacteria from the environment. In contrast to the high taxonomic diversity, the changes of PICRUSt inferred function profile were minimal for all cowpats throughout the study, which suggest that core functions predicted by PICRUSt may be too conserved to distinguish differences between aerobe and anaerobe. To the best of our knowledge, this is the first study demonstrating that cowpat exposure to air and sunlight can cause drastic microbiome changes soon after deposition in natural environments. Our findings offer important insights for future research characterizing the microbiome of feces collected in natural environments and the impact of cattle fecal contamination on water resources.

## Introduction

Fecal pollution of environmental waters is a major concern for the public because exposure to fecal-associated pathogens can have severe impacts on human health (Maier et al., 2008). Animal shedding of feces is a major source of fecal contamination, particularly in agricultural areas where animal manure is land-applied without prior treatment or where farm animals have direct access to surface waters, as is the case for some animal feeding operations. Detection of fecal indicators, source tracking markers and pathogens using molecular techniques such as PCR and quantitative PCR (qPCR) has increased substantially in the last couple decades (Kong et al., 2002; Scott et al., 2002). In addition, the recent adoption of next generation sequencing (NGS) has facilitated greater characterization and understanding of the composition and structure of the fecal microbiome (Nakamura et al., 2009; Looft et al., 2012; McLellan et al., 2013; Newton et al., 2013), which is critical to fecal pollution detection as well as animal health and food safety (Shanks et al., 2011).

A majority of metagenomic studies on fecal microbiomes using NGS, focus on fresh feces (Unno et al., 2010; Shanks et al., 2011; Looft et al., 2012; Cao et al., 2013; Ye and Zhang, 2013). However, very little is known about how the community structure of fecal bacteria changes after shedding into the environment. Previous studies showed that the community structure of fecal bacteria is sensitive to environmental stresses induced by engineering and human intervention such as sewage treatment, diet, antibiotic intake and nanoparticles exposure (Looft et al., 2012; Ye and Zhang, 2013; Yang et al., 2014). After animal shedding or land application of manure, natural environmental stressors such as sunlight and moisture level of feces strongly influence the survival of bacteria (Natvig et al., 2002; Sinton et al., 2007; Oladeinde et al., 2014), and can potentially influence the composition and ecology of the fecal microbiome. Furthermore, previous survival studies showed that different fecal bacteria have different re-growth and decay patterns in the environment (Crane et al., 1980; Davies et al., 1995; Oladeinde et al., 2014). Therefore, after exposure of freshly excreted feces to the environment, the abundance of different fecal bacterial groups is expected to vary. There is very limited knowledge in this area and no study has yet investigated the progressive change of the fecal bacterial community structure resulting from exposure to environmental conditions.

The main objectives of this study are to understand (i) the progressive change of the community structure in freshly excreted cowpats under field conditions and (ii) the impact of sunlight exposure, with correlated changes in moisture content, on the cowpat microbial community structure. Core functional profiles and the abundance of genera consisting of pathogenic genera are also included in this study. This type of information will be very useful for future fecal pollution research such as identification of better microbial indicators and pathogen detection through the aging process. We focus on cattle feces because agricultural activities and livestock are the greatest contributors of fecal pollution to streams and rivers in the U.S (Guber et al., 2007; USEPA, 2009).

## Materials and Methods

### Manure Collection, Study Site, and Sample Collection

Detailed procedures for manure collection and study site description can be found in Oladeinde et al. (2014). Briefly, a total of eight freshly excreted bovine cowpats were used in this study. One cowpat per cow, four cows per farm were collected from two beef-producing farms in north Georgia during the summer of 2012. Both farms were privately owned and permissions to collect fecal samples were given by the owners of the farms. Farm #1 was located in Jackson County, GA, USA, while Farm #2 was located in Madison County, GA, USA. Following excretion, cowpats were collected as whole as possible, using 8-inch-diameter, 24-gage round-end stove caps (Grainger Inc., Lake Forest, IL, USA) and a 24-inch × 12-inch piece of sheet metal-gage steel (Stanley Hardware, New Britain, CT, USA). The round stove cap end was placed carefully on the cowpat to avoid disturbing its original structure as much as possible. Cowpats were immediately transported on ice and in the dark to the study site located at the US EPA Ecosystems Research Division in Athens, GA, USA. Cowpat weights ranged from 0.7–1.6 kg (Supplementary Table 1). Two cowpats from each farm were exposed to sunlight and two others were shaded by placing a solid-color tarp over them. Plot covers (2.4 m × 1.7 m) were constructed of PVC frames lined with clear acetate films (Grafix Plastics, Cleveland, OH, USA; 80% UV-transmission) and placed on top of each treatment set to protect the cowpats from natural rain events. One additional cowpat was fitted with a 12–Bit Smart temperature sensor connected to an onset HOBO U30 data logger (Onset Computer Inc., Bourne, MA, USA). A UV sensor (Satlantic model OCR-504) measuring four different wavelengths (305, 325, 340, and 380 nm) was installed underneath both treatment plot covers and connected to a Stor-X data logger (Satlantic, Halifax, NS, Canada). One additional UV sensor was installed away from the plot covers to monitor full sunlight.

Four cores of fecal samples were collected on days 0, 2, 4, 6, 8, 15, 22, 29, 43, and 57, between 9:00 and 10:00 am from each cowpat; cores were collected from both the outer crust and moist interior of the cowpat to obtain representative samples of the entire cowpat. Cores sampled from each cowpat were homogenized to yield a total of eighty samples (10 per cowpat) collected for sequencing analysis. The moisture content of each homogenous fecal sample was determined gravimetrically by drying 2–5 g at 105°C until equilibrium was reached.

### DNA Extraction

One hundred milligram of each homogenized cowpat sample were transferred to PowerBead tubes in triplicate (MoBio Laboratories, Carlsbad, CA, USA) and stored at -80°C until extraction, which occurred within 2 weeks of sampling. DNA was extracted using the MoBio Power-Soil DNA Isolation kit following the manufacturer’s instructions.

### PCR Amplification

Two sets of fusion primers were used: the first set generated the primary 16S amplicons and the second converted the primary amplicons into libraries for sequencing. Primers targeting 16S rRNA hypervariable V4 region were selected to capture the diversity of fecal-associated bacteria (Forward: 5′-CAGCMGCCGCGGTAATWC-3′; Reverse: 5′-CCGTCAATTCCTTTRAGGTT-3′; Lane et al., 1985). Forward primers also contained the Illumina Nextera Read 1 sequence (5′-TCGTCGGCAGCGTCAGATGTGTATAAGAGACAG-3′), whereas reverse primers had the Illumina Nextera Read 2 sequence (5′-GTCTCGTGGGCTCGGAGATGTGTATAAGAGACAG-3′). Unique sequence tags of varying lengths (5–8 nt) were synthesized between the Nextera and 16S sequences (Supplementary Table 2). Second round PCRs used iNext i5 (5′-AATGATACGGCGACCACCGAGATCTACAC NNNNNNNN TCGTCGGCAGCGT^∗^C-3′) and iNext i7 (5′-CAAGCAGAAGACGGCATACGAGATNNNNNNNN GTCTCGTGGGCTCG^∗^G-3′) primers where the 8N’s are replaced by the specific tag sequences given in Supplementary Table 2 and the ^∗^ designates a phosphorothioate bond. A total of eight forward and 12 reverse fusion primers were synthesized. For the first PCR round, three independent 25 μl PCR reactions were run using the following conditions: initial denaturation at 98°C for 30 s, and 22 cycles at 98°C for 7 s, 55°C for 30 s, 72°C for 15 s and a final extension at 72°C for 7 min. To set up the second PCR, the triplicate PCR reactions were combined and run using conditions similar to the first round of reactions, except there were only 10 amplification cycles. Each PCR reaction mix included 12.5 μL of 2X Phusion^®^ High-Fidelity PCR master mix; 1.0 μL of each forward and reverse primer (each final concentration was 400 nM); 8.5 μL of PCR-grade water and 2 μL of DNA sample. The concentrations of amplicons from different samples were normalized using a SequalPrep Normalization Plate Kit (Invitrogen, Carlsbad, CA, USA). After normalization, all samples were pooled and sequenced at the Georgia Genomics Facility using an Illumina MiSeq v2 600 cycle kit. A 300 bps paired-end sequencing reaction was performed on a MiSeq platform (Illumina, San Diego, CA, USA). PCR products of ∼550 bp (paired-end insert size) were obtained for all 80 samples. Of the 550 bp, ∼400 bp represented the 16S amplicons (including the primers). Seven samples with few reads were resequenced. Reads were submitted to NCBI SRA under accession SRP064539 and bioproject PRJNA297907.

### Bioinformatics Analysis

Reads with a quality score less than 15 were trimmed using fastq_quality_trimmer from the FASTX toolkit version 0.0.13.2 (FASTX-Toolkit, 2014). Forward and reverse barcode combinations were used to identify each sample. Paired-end reads were joined together using fastq-join from ea-utils.1.1.2-537 (Aronesty, 2011), and unjoined reads were filtered out by an in house script. SeqFilters.jar was used to extract sequences between V4 region primers (Maidak et al., 1996), and chimeric reads were filtered out using UCHIME with the “Gold” database (Edgar et al., 2011). The final high quality reads were analyzed using QIIME version 1.8.0 under default parameters (Caporaso et al., 2010). The operational taxonomic unit (OTU) picking was performed using UCLUST at a 3% threshold (Edgar, 2010) and the OTUs with fewer than 10 reads were removed to avoid PCR sequencing errors. OTU taxonomy was assigned by RDP 2.2 at a confidence level cutoff of 80% (Wang et al., 2007). The relative abundance (RA), as well as temperature and moisture reported here, is the averaged RA of two cowpats. U1, U2 and S1, S2 are the abbreviations for unshaded (U) and shaded (S) samples from Farm 1 and 2, respectively. Non-metric multidimensional Scaling (NMDS) based on the Bray–Curtis dissimilarity matrix was performed using the R-VEGAN package in R^1^ to visualize the community structure. Both replicate samples were plotted on NMDS to visualize their structural differences. Once the iterative NMDS solution is found, the R-VEGAN package uses a random permutation method (“envfit”) for determining whether factors and covariates are significantly influencing the NMDS scores. For categorical factors (unshaded and shaded fecal samples), “envfit” uses the difference between the average NMDS scores (centroids) at each level of the factor. Since significant differences were found among different factors (see “Results” section), envfit was also used to determine if there is a significant difference between two individual centroids. For a covariate (moisture), the test compares the length of the fitted vector on the NMDS axes to a distribution of fitted vector lengths calculated after randomly permuting the dataset. Ten-thousand permutations were run to construct the distributions of fitted vector lengths and factor-level differences. We defined a *p*-value (*p*) less than 0.05 as a significant difference for factors and covariates.

Alpha diversity analysis was calculated via a rarefaction curve from QIIME’s alpha diversity pipeline (Caporaso et al., 2010). Duplicate samples were combined and normalized to 10.5K reads, which was equivalent to the sample with the least reads, for estimating species richness (Chao1) and diversity (Shannon index). Evenness was calculated by dividing the Shannon index by the natural log of Chao1.

In addition to community structure analysis, Phylogenetic Investigation of Communities by Reconstruction of Unobserved States (PICRUSt) was also used to infer the functional content, based on 16S OTU BIOM generated from QIIME (Langille et al., 2013). The RA of PICRUSt inferred function is also reported here.

To determine significant differences in RAs, simple *t*-tests were performed using R; a *p*-value (*p*) of ≤0.05 indicates a significant difference.

## Results

### Moisture, UV Intensity, and Temperature

As expected, UV intensity and temperature of unshaded samples was higher than shaded samples (Supplementary Figure 1). The average UVA and UVB difference between unshaded and shaded sites throughout the study was 1.94 ± 0.21 μW cm^-2^ and 40.20 ± 4.97 μW cm^-2^, respectively. The temperature difference between unshaded and shaded cowpats ranged from ∼2 to 5°C. The moisture content of all samples was similar at day 0 and slowly declined until day 8. U2 had a more rapid decline of moisture content than other samples, at least 15% lower by day 8. From days 0 to 8, more rapid decline in moisture for U2 and S1 than U1 and S2 may be due to lighter average weights of U2 and S1 (Supplementary Table 1). The duplicate cowpats of S1 weighted very differently (Supplementary Table 1) but their moisture levels were similar except on days 15 and 22 (Supplementary Figure 1), where the moisture levels of S1#1 were 60 and 55% higher than S1#2 on days 15 and 22, respectively. After day 8, all samples experienced a rapid decline in moisture content, but it was faster for unshaded samples. By the end of the study, moisture content of unshaded samples was slightly lower (∼5%) than in shaded samples. Moisture and sample day had a significant inverse correlation based on linear regression analysis (*p* < 0.05).

### PCR Products and Illumina Sequences

Illumina MiSeq 300 PE sequencing of the 80 samples gave a total of 8,842,490 reads. After fastq quality trimming, pair-end joining, and barcode and chimeric filtering, 2,490,514 high quality joined reads were obtained and used for downstream analyses. Individual samples that passed QC generated 8.7–43.5K reads. Samples that failed initial QC were re-sequenced, generating 23–127K reads with average reads per sample being 64K. After clustering and taxonomic assignment with a 3% cutoff, OTUs with less than 10 observation counts were removed. Finally, the number of OTUs for individual samples ranged from 2.1 to 8.3K, with an average of 5.2K.

### Composition of Bacterial Community at Phylum and Class Level

Supplementary Figure 2 illustrates the RA of cowpats at the phylum level. Throughout the entire study, *Actinobacteria, Proteobacteria, Firmicutes*, and *Bacteroidetes* were the major phyla detected, with almost 90% of the bacteria assigned to these phyla. The major phyla at day 0 were *Firmicutes* (44–57%) and *Bacteroidetes* (35–49%), but their RA decreased to <19% by day 57 (*p* < 0.05). In contrast, *Actinobacteria* and *Proteobacteria* increased for both shaded and unshaded samples over time, from less than 1% at day 0 to at least 65% by day 57. All four phyla had significantly different abundance in days 0 and 57 samples in both sets of shaded and unshaded samples (*p* < 0.05).

**FIGURE 1:**
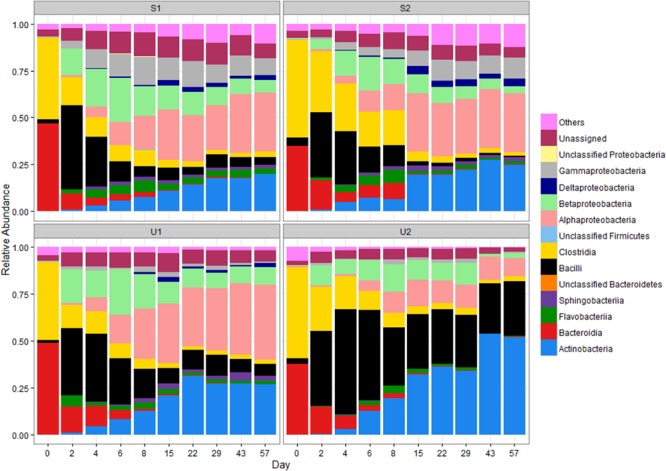
**Relative abundance (RA) of bacterial classes in shaded (S) and unshaded (U) samples from farm 1 and 2.** The RA is calculated by averaging abundances of two individual cowpats. “Others” includes all classes with less than 0.5% RA. NA, not assigned.

The RAs at the class level of the four major phyla are illustrated in **Figure 1**. The RA changes of two major classes of *Firmicutes* are very different: *Clostridia* declined over time (from ≥44 to ≤3%) while *Bacilli* abundance increased significantly from days 0 to 2 in all samples (from ≤5 to ≥34%; *p* < 0.05). This increase was followed by a gradual decrease after day 2 for a final RA of ≤6%. In contrast, the final RA of *Bacilli* was much higher for U2 (∼29%). The RA of *Clostridia* for S2 declined more gradually than other cowpats but both shaded and unshaded samples ended with ≤3% of *Clostridia* by day 57, and became significantly lower than day 0 (*p* < 0.05).

*Bacteroidia* was the major class of *Bacteroidetes* in day 0 cowpats (**Figure 1**), but experienced a gradual decline throughout the study to a final RA of ≤ 0.5% (*p* < 0.05); in contrast, the RA of *Flavobacteria* increased from day 2 to 8 especially in the shaded cowpats. The RA of *Actinobacteria* for U1, S1, and S2 was between 19 and 27% by day 57; however, it was more than 30% for U2 by day 22 and close to 52% by day 57.

The RA of *Alphaproteobacteria* increased throughout the study and it was the most prevalent *Proteobacteria* by day 57. Even though the RA of *Betaproteobacteria* was higher than that of the *Alphaproteobacteria* from day 2 to 6, the *Betaproteobacteria* RA decreased gradually after day 8 and became lower than the *Alphaproteobacteria* abundance in all cowpats by day 15. Despite U1, S1, and S2 having similar RAs of *Alphaproteobacteria* throughout the study, the RA of *Alphaproteobacteria* in U2 was lower than that of the other samples from day 15 to 57 and its abundance at day 57 was significantly lower than other three samples (*p* < 0.05). The RAs of *Gammaproteobacteria* and *Deltaproteobacteria* were lower (combined RA ≤ 3%) than other *Proteobacteria* in unshaded cowpats, while they were more abundant in shaded samples (combined RA ≥ 11%), especially *Gammaproteobacteria* (RA ≥ 8%).

**FIGURE 2:**
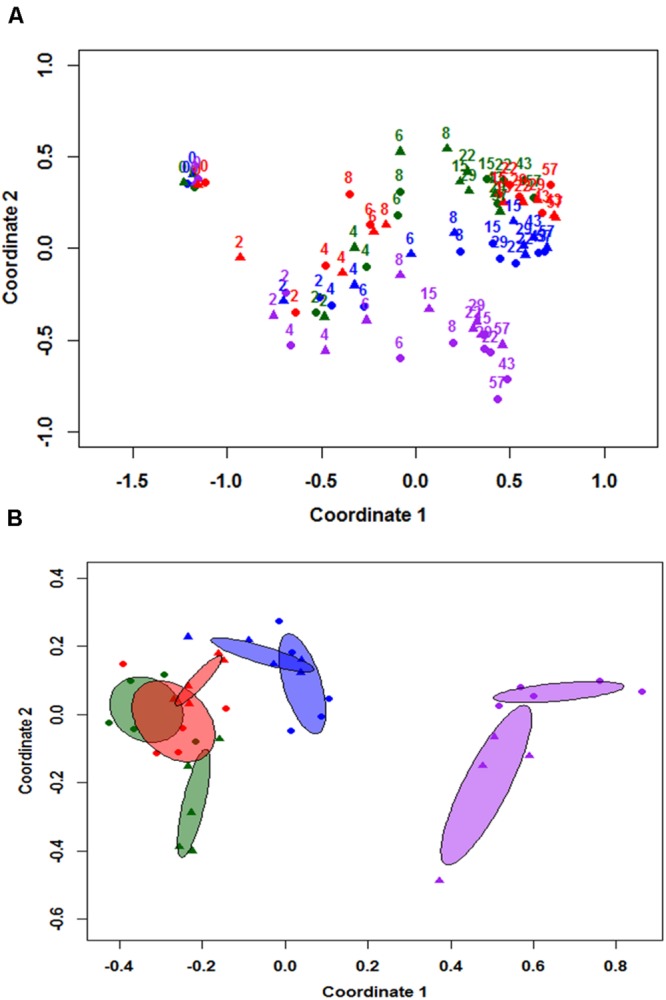
**Non-metric multidimensional scaling (NMDS) analysis based on community structure at class level **(A)**, and 95% centroids of days 15–57 samples **(B)**.** Numbered labels in figure **(A)** represent sampling day and colors apply to both figure **(A,B)**. Blue = duplicate cowpats from unshaded farm 1 (U1), Purple = duplicate cowpats from unshaded farm 2 (U2), Green = duplicate cowpats from shaded farm 1 (S1), Red = duplicate cowpats from shaded farm 2 (S2). Circle (•) and triangle (▴) symbols represent each duplicate cowpat.

NMDS results were used to differentiate the community structure of all eight individual cowpats at the class level as illustrated in **Figure 2A**. Results indicate the trends of community structure changes of duplicate samples throughout the study were similar and majority of duplicate samples clustered together. At day 0, the community structure of the four unshaded and the four shaded samples grouped together. A continuous change of community structure from days 2 to 8 can be inferred based on all eight samples shifting toward the right side of the NMDS plot. Despite the large difference in moisture levels of duplicate S1 cowpats on days 15 and 22, their community structure differences on both days were minimal and they clustered together on NMDS plot (**Figure 2A**). The distribution of samples from day 15 to 57 is illustrated with the centroid plots in **Figure 2B**. Both U2 centroids (purple centroids) are distinct from other centroids, which is mainly due to the higher RA of *Bacilli* and *Actinobacteria* and lower RA of *Alphaproteobacteria* relative to the other six centroids. Despite the close proximity of duplicate centroids in **Figure 2B**, the envfit analysis showed that all eight centroids are significantly different from each other (*p* < 0.05), including the four S1 and S2 centroids (*p* = 0.024). In addition, the length of the fitted moisture vector is significantly greater than the randomly permuted fitted moisture vector length, indicating that sample points in NMDS space are significantly correlated to moisture.

### Family Compositions of *Alphaproteobacteria, Betaproteobacteria, Actinobacteria*, and *Bacilli*

The RAs at the family level were used to further investigate the community structure of *Alphaproteobacteria, Betaproteobacteria, Actinobacteria*, and *Bacilli*, which were the dominant classes by the end of the study.

Members of the family *Coriobacteriaceae* were the most dominant *Actinobacteria* at day 0; however, *Coriobacteriaceae* RA decreased significantly in all samples after day 0 (*p* < 0.5; Supplementary Figure 4). *Nocardiaceae, Nocardioidaceae, Mycobacteriaceae*, and *Patulibacteraceae* became the dominant families of *Actinobacteria* in all samples by day 57.

Unclassified *Alphaproteobacteria* and RF32 dominated the *Alphaproteobacteria* communities in all samples (≥90% of their combined RA) at day 0; however, the RA in all samples significantly decreased to a range of ≤0.3% by day 57 (*p* < 0.05; Supplementary Figure 4). The RA of *Sphingomonadaceae* for all samples also increased after day 0 but began to decrease by day 6, and the increase in *Sphingomonadaceae* abundance was larger for unshaded samples. Despite the difference between samples, *Bradyrhizobiaceae, Caulobacteraceae, Hyphomicrobaceae, Phyllobacteriaceae, Rhizobiaceae, Rhodobacteraceae*, and unclassified *Rhizobiales* became the dominant *Alphaproteobacteria* families in all samples by day 57 with a combined RA ranging from 78 to 88%.

Unclassified *Betaproteobacteria* and *Alcaligenaceae* were the dominant *Betaproteobacteria* at both order and family levels at the beginning of the study (Supplementary Figure 5). *Comamonadaceae* and unclassified *Burkholderiales* were the two most dominant groups at day 2 for all samples and the community structure for shaded samples remained relatively similar throughout the study. The RA of *Alcaligenaceae*, however, gradually increased and eventually became the dominant *Betaproteobacteria* family again in unshaded samples. The RA of *Oxalobacteraceae* gradually increased in all samples but more substantially in the unshaded samples. The RA of *Methylophilaceae* increased only in shaded samples.

*Planococcaceae* was the most dominant *Bacilli* in all samples during the study. The RA of *Bacillaceae* also remained stable throughout the study. *Paenibacillaceae* increased substantially in most samples (Supplementary Figure 6).

### Composition of Pathogenic Bacteria at Genus Level

**FIGURE 3:**
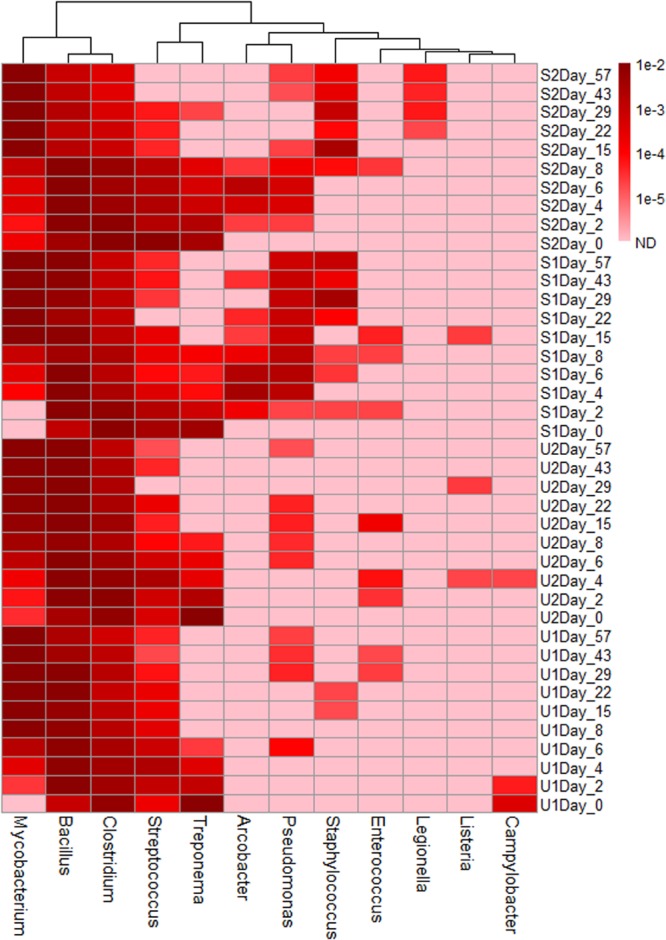
**Heatmap of genera consist of pathogenic species.** The abundance scale of heatmap ranged from 10^-2^ to non-detected (ND). Pink color in the heat map indicates ND.

Based on the previously reported genera consisting of pathogenic fecal bacteria (Ye and Zhang, 2011), 12 genera were identified in our samples; their total abundance throughout the entire study varied from 1.6 to 4.9% (Supplementary Figure 7). Three genera, *Mycobacterium, Bacillus*, and *Clostridium*, were the most abundant; with a combined abundance of 90% of the total pathogenic genera. Genera such as *Mycobacterium* had a higher abundance in aged feces, but others such as *Clostridium, Streptococcus*, and *Treponema* had higher abundance in fresh feces (**Figure 3**). The presence of some genera was also treatment-specific: *Staphylococcus, Arcobacter*, and *Pseudomonas* were present more often in shaded samples than in unshaded.

### Alpha Diversity Analysis

**FIGURE 4:**
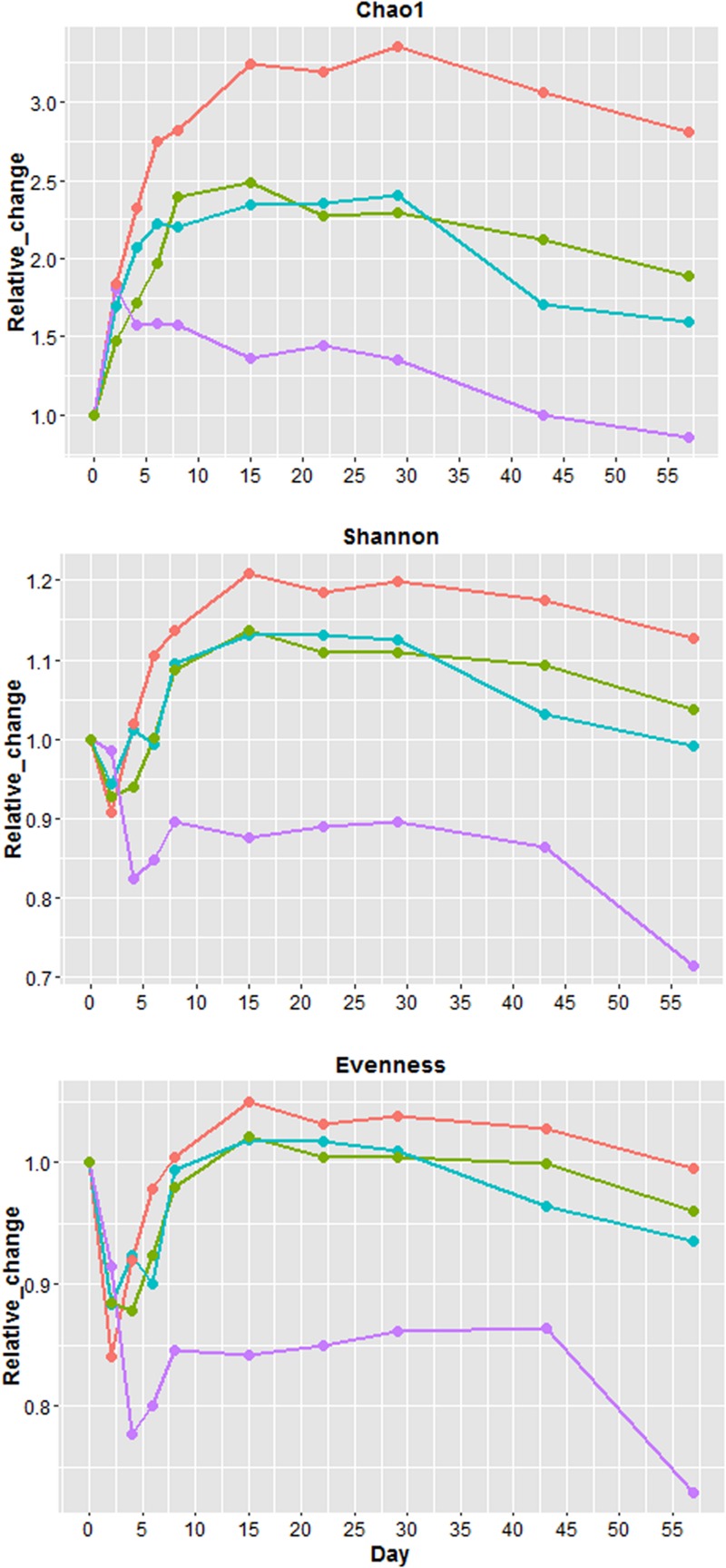
**Relative changes in Chao1 (richness), Shannon index (diversity) and evenness.** Relative change = (value of each sampling day)/(value of day 0). Blue = unshaded farm 1 (U1), Purple = unshaded farm 2 (U2), Green = shaded farm 1 (S1), Red = shaded farm 2 (S2).

Because all four samples had different values of Chao1 (richness), Shannon (diversity) and evenness at day 0 (Supplementary Figure 8), the values from each sampling days were normalized (**Figure 4**) by day 0 values to illustrate the relative changes overtime. Overall U1, S1, and S2 richness increased up to day 15 followed by a plateau phase, and decreased after day 29 (**Figure 4**). For U2, richness only increased from day 0 to 2 followed by a gradual decline until day 57. After day 0, S2 had the greatest increase in richness, while U2 exhibited the smallest increase. The changes of richness for U1 and S1 were relatively similar throughout the study. Except for U2, other samples at day 57 had a higher richness than day 0. Shannon diversity index decreased in all samples at the beginning of the study with U2 having the greatest reduction (∼18%). For U1, S1, and S2, the diversity increased substantially after day 4 and it became 13 to 21% higher than day 0. For U2, the diversity only increased slightly after day 4 and it remained at least 10% lower than the diversity at day 0. Similar to diversity, the evenness also declined at the beginning of the study with U2 having the greatest reduction (∼23%). Overall, S2 has the greatest increase of richness, diversity and evenness throughout the study, the changes of these factors were similar between S1 and U1, and the index values of these factors was the lowest for U2 throughout the study.

The number of OTUs in the rarefaction curves with a 3% cutoff did not level off by day 57 (Supplementary Figure 9), indicating that additional sequencing depth is needed for sufficient species coverage. At 5% cutoff (Supplementary Figure 10), all samples still needed more sequence reads to reach the plateau but sequence depth was almost sufficient for aged (days 43 and 57) U2 samples. The number of OTUs continuously increased from day 0 to day 15 in U1, S1, and S2 samples, but in U2 samples, only days 2 and 4 had higher numbers of OTUs than day 0.

### Functional Analysis

**FIGURE 5:**
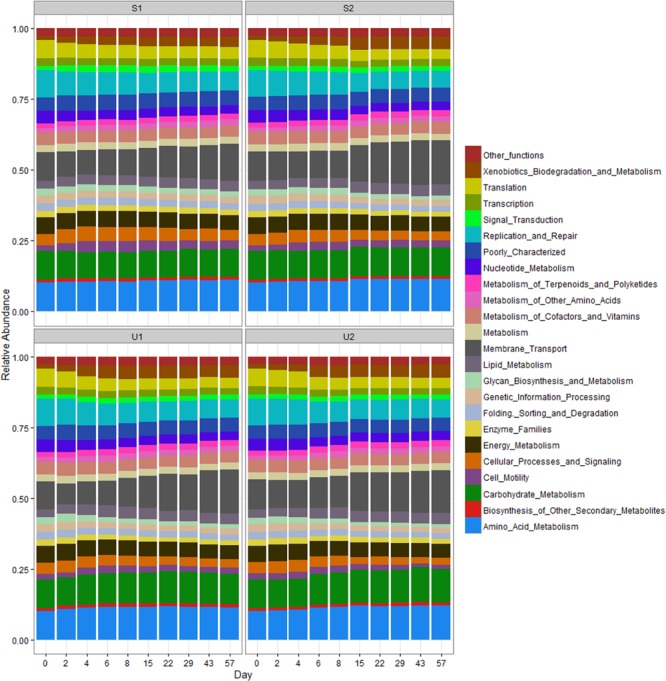
**Relative abundance of PICRUSt inferred function in shaded (S) and unshaded (U) samples from farm 1 and 2.** The RA is calculated by averaging the abundances of duplicate cowpats. “Other functions” includes all inferred functions with less than 1% RA.

The RA of PICRUSt inferred function is illustrated in **Figure 5**. Compared to taxonomic profiles, the functional profiles of all samples were much more similar to each other. Nevertheless, a few functional changes were observed over time, regardless of UV exposure or farm location. For instance, all cowpats had an increase in the abundance of membrane transport and xenobiotic biodegradation and metabolism but a decrease in the abundance of translation, replication and repair, and cellular processes and signaling throughout the study. Differences of all these functions between days 0 and 57 were significant (*p* < 0.05). Amino acid and carbohydrate metabolism, membrane transport and replication and repair were the most abundant functions in all samples.

Since aerobic bacteria is more effective in generating energy than anaerobic bacteria, different pathways in energy metabolism were compared. The RA of energy metabolism and carbon fixation at days 0 and 2 was significantly higher than the rest of samples (*p* < 0.05), but no significant difference was found between day 0 and day 2 samples (*p* > 0.05; Supplementary Figure 11). The RA of methane metabolism at day 0 was significantly higher than other samples (*p* < 0.05). There were no significant difference in both nitrogen and sulfur metabolisms among the samples collected at different dates (*p* > 0.05). The mean RAs of energy metabolism, methane metabolism and carbon fixation were reduced by 27, 27, and 13%, respectively, between day 0 and day 57 samples.

## Discussion

This paper reports application of high throughput sequencing to determine changes of the cattle fecal microbiome over time after release into the environment. Results clearly indicate that the community structure of fecal bacteria after deposition to the environment changes drastically within just 2 days. Two of the most dominant classes of bacteria at day 0, *Clostridia* and *Bacteroidia* are obligate anaerobes (Baron et al., 1996; Coyne et al., 2013), and exhibit an initial combined RA of 86 to 90%, but their combined RA decreased to only 2 to 3% by day 57. On the other hand, the RAs of *Alphaproteobacteria, Betaproteobacteria, Actinobacteria* in all cowpats, *Bacilli* in U2, and *Gammaproteobacteria* in shaded cowpats increased throughout and/or by the end of the study. The family members of *Alphaproteobacteria* that increased substantially throughout the study are all aerobic bacteria and belong to the *Acetobacteraceae, Methylobacteriaceae*, as well as four major nitrogen fixing bacterial groups, *Rhizobiaceae, Bradyrhizobiaceae, Hyphomicrobiaceae*, and *Phyllobacteriaceae* (Ivanova et al., 2001; Gallego et al., 2006; Greenberg et al., 2006; Clark et al., 2009). The most dominant family of *Actinobacteria* at day 0, *Coriobacteriaceae* (Harmsen et al., 2000), are obligate anaerobes. In contrast, *Nocardiaceae, Nocardioidaceae, Mycobacteriaceae*, and *Microbacteriaceae* are aerobes (Lechevalier et al., 1977; Stackebrandt et al., 1997; Han et al., 2003) and were the four most abundant *Actinobacteria* present by day 57. In addition, *Clostridia* had a rapid decline in RA by day 2 while *Bacilli*, which have the ability for aerobic respiration (Nazina et al., 2001), increased substantially. Therefore, a major trend of the fecal microbiome in this study for all samples is a community structure shift from anaerobic to aerobic bacteria. Although this observation has not been reported in previous studies to the best of our knowledge, the microbiome results are consistent with the large oxygen level difference between animal gut and open field environment. Also, if diet, host origin and antibiotic usage can influence the community structure of the fecal microbiome (Unno et al., 2010; Shanks et al., 2011; Looft et al., 2012), why wouldn’t microbiome change substantially when expose to an environment with a completely different oxygen level?

We believe the reduction in moisture level in the cowpat increased the exposure of cowpats to oxygen/air due to air being able to diffuse more readily into the pore space of drier feces. In addition, moisture is significantly correlated with sampling date, and the directional change of the community structure is significantly correlated to moisture based on the envfit analysis. Therefore, moisture content of cowpats could be a factor influencing the microbial diversity of feces deposited in the field environment. However, we cannot argue that moisture is the only important factor influencing community structure in all cases. For instance, a minimal difference was identified in duplicate S1 cowpats on day 15 and 22 while their moisture content was drastically different. Since oxygen inside the cowpats was not measured in this study, we recommend measuring this factor in future studies to determine correlations between moisture and oxygen level inside the cowpats, and between oxygen level and abundance of the aerobe/anaerobe community.

Our data suggest that sunlight is affecting the microbial diversity based on the fact that the community structures differ between unshaded and shaded cowpats, especially those collected at farm 2. Our previous study also showed that the re-growth and decay pattern of different fecal indicators was not the same in shaded and unshaded samples (Oladeinde et al., 2014). Exposure to sunlight resulted in higher UV intensity and temperature and faster loss of moisture content in unshaded cowpats (Supplementary Figure 1); however, which factor has more influence on community structures could not be determined here. In addition, there is a greater separation between the centroids of shaded and unshaded cowpats than the centroids of duplicate cowpats in NMDS plots, indicating that the differences between community structures of shaded and unshaded samples by the end of the study were not due to variation in duplicate samples and/or measurements but were induced by sunlight. Also, considering the difference between unshaded samples (U1, U2) is much greater than the difference between shaded samples (S1, S2), it indicates that feces from different farms under the sunlight exposure can result in different microbial communities and therefore animal history such as diet should also be taken into consideration. Previous studies have shown that photodegradation of organic matter can have significant impact on bacterial growth (Lindell et al., 1995; Tranvik and Bertilsson, 2001; Fasching and Battin, 2012); therefore, if the composition of organic matter in the feces from different farms change after photodegradation, it can strongly influence the growth and decay pattern of bacteria, thus affecting the community structure.

This study showed that the RAs of certain bacterial groups (e.g., *Bacilli* and *Betaproteobacteria*) did not exhibit the monotonic trend that *Alphaproteobacteria* and *Bacteroidia* did, but instead their RAs increased initially and then decreased (**Figure 1**), indicating that the environmental conditions favoring the abundance of these bacteria changed over time. This could be due to different fecal bacteria having different decay or re-growth rates in the environment and thus affecting the RA of individual groups in the community (Crane et al., 1980; Sinton et al., 2007; Oladeinde et al., 2014). For example, increase of *Bacilli* abundance from days 0 to 2 in all samples can be caused by an increase in the concentration of *Bacilli*, but also could be due to a reduction in the abundance of *Clostridia*. Future studies should determine if a change of RA correlates to changes of absolute bacterial concentration by including qPCR analysis for absolute quantification of different bacterial groups.

Contrary to the high taxonomic changes observed in our data, the RA of PICRUSt inferred functional profiles remained fairly consistent throughout the study. A similar observation was also reported in a human microbiome study where distinct differences in taxonomic structure were found among the samples collected from different body habitats, but the abundance of metabolic pathways remained consistent among different samples. A previous study used both shotgun metagenomics and PICRUSt prediction to determine the core bacterial functions from different Upper Mississippi River water samples, and results from both approaches also showed that function profiles were more consistent than the 16S taxonomic community profiles (Staley et al., 2014). Such consistency could be due to different bacteria having similar core functions, but it could also indicate that the core functions predicted by PICRUSt are too conserved to distinguish differences between aerobes and anaerobes. For example, PICRUSt can only provide the abundance of methane metabolisms, but there are four modes of methane metabolism that include aerobic and anaerobic processes (Oremland, 2010). Higher coverage using shotgun metagenomics may therefore be needed to have a finer resolution of bacteria function profile such as differentiating various methane metabolism modes.

A significant decrease in the energy metabolism function may be due to shifting from an anaerobic to an aerobic bacterial community. Decreased overall energy metabolism throughout the study may be a results of the decrease of anaerobic bacteria observed in our samples. A higher abundance of energy metabolism function may be needed to sustain the anaerobic community because energy production by anaerobe is less efficient than by aerobe. Consistency in nitrogen and sulfur metabolism throughout the study could be due to both aerobic and anaerobic bacteria having these two metabolisms. A significantly higher abundance of methane metabolism in day 0 was most likely due to the present of methanogens (*Methanobrevibacter* and *Methanosphaera*), which are anaerobic bacteria and they were detected at day 0 samples more often than other samples (data not shown).

Diversity is a function of richness and evenness. Because evenness in all four samples increased to days 8 (U2) and 15 (U1, S1, S2) and stayed at plateau until day 29, no samples during this period had a decline of diversity – even U2, whose diversity remained relatively constant despite continuous decline of richness. Richness, diversity, and evenness of all samples began to decrease around day 29. A couple of possible reasons may account for the increase of overall richness and diversity. One possible reason is the growth of low abundance bacteria to detectable levels, such as the re-growth of *Proteobacteria* and *Actinobacteria*. Another possible reason is the colonization of airborne bacteria from the field environment (e.g., from surrounding soils) since cowpats were placed in the metal containers with no top cover throughout the study. Evidence of this is the fact that *Nocardiaceae*, commonly found in soils, was one of the most dominant *Actinobacteria* families found in our samples as the study progressed. Because no soil or air samples were sequenced in this study, we do not know the level of influence by surrounding soil bacteria on the community structure of cowpats.

Based on the rarefaction curves from this and previous studies, more sequence reads are needed for sufficient coverage for cattle feces in future studies. Even with 80K reads, the rarefaction curves for most cattle feces were not able to reach plateau at 3% cutoff (Shanks et al., 2011). Unno et al. (2010) and Jeong et al. (2011) compared rarefaction curves of feces from different animals and found both beef and dairy cattle feces had the highest number of OTUs; rarefaction curves of cattle feces in both studies were also not able to reach plateau at 3% cutoff.

There has been increased interest in identifying better microbial signatures of fecal-associated samples, using NGS, to improve water quality monitoring (Unno et al., 2010; Shanks et al., 2011; Newton et al., 2013; Ye and Zhang, 2013). Applications in these studies focused on identifying fecal-associated pathogens and fecal signature bacteria for microbial source tracking. We believe the results reported here provide useful insights for future studies in applying community analysis to monitor and track fecal water pollution. The fecal microbiome affecting surface water in the agricultural areas originates from both fresh and aged feces, however, previous studies have focused mostly on identifying the relationship between the microbiome of polluted water and fresh feces. We therefore believe they viewed the fecal microbiome, more or less, as a “static” microbiome without recognizing possible dynamic changes after feces are released from mammal gut into field environment. Fecal pollution can easily be overlooked if only microbiome information of fresh feces is used to identify the pollution or its source.

Another major application of NGS to water quality is pathogen detection. We were able to detect 13 genera consisting of pathogenic bacterial species. Similarly to observations in phylum, class, and family classification, the genera that are present more abundantly in fresh/slightly aged feces (*Clostridium, Streptococcus*, and *Treponema*) are anaerobe, but the genera that is present more abundantly in aged feces (*Mycobacterium*) are aerobe. In addition, *Bacillus* can be aerobic or facultative anaerobic, and were detected in both fresh and aged feces. Therefore, detection of pathogenic bacteria in feces and fecal-impacted water can be influenced by feces freshness. Our results also show sunlight irradiation might reduce chances of pollution due to certain bacterial pathogens such as the pathogenic species in *Staphylococcus, Arcobacter*, and *Pseudomonas*. *E. coli* was detected using qPCR in our previous study (Oladeinde et al., 2014), however, no *Escherichia* was detected in this study, which is likely due to the fact that our study did not have enough reads covering all the genera since the rarefaction curves did not plateau. To improve the reliability of using NGS for fecal pathogen detection, it is therefore important for future studies to determine numbers of reads required for covering all genera and species in different fecal samples.

There has been increasing attention to study fecal microbiome for different purposes, however, many of these studies do not mention the level of freshness of fecal samples during collection. Although our intention was not to investigate the influence of the fecal sampling procedure on community structure, results highlight importance of fecal sample freshness because exposure to air and sunlight for even a short period can have a significant impact on the fecal bacterial profile. When studying factors such as diet, treatment received or host origin on fecal microbiomes, it is important to collect fresh samples to maintain integrity of the microbial community structure as it exists in the feces before shedding. This is emphasized by the large differences on the fecal bacterial community structure observed between days 0 and 2. To gain a better understanding of the lasting impact of fecal microbiome on environmental/surface waters, it is important for future research to cover a more comprehensive timeframe, without losing perspective of the rapid changes occurring shortly after deposition.

## Author Contributions

KW, MM, TS, AO, TG, and BO conceived and designed the research project, and contributed with interpretation of results. KW, MM and TS wrote the manuscript and performed the statistical analysis. TS and KW conducted the bioinformatics analysis. KW and AO performed the laboratory analysis.

## Disclaimer

This report has been subjected to the U.S. EPA’s peer and administrative review and has been approved for publication. The mention of trade names or commercial products in this report does not constitute endorsement or recommendation for use.

## Conflict of Interest Statement

The authors declare that the research was conducted in the absence of any commercial or financial relationships that could be construed as a potential conflict of interest.

## Footnotes

1 http://www.r-project.org/
