# Supplementary material for: Rapid Microbiome Changes in Freshly Deposited Cow Feces under Field Conditions
Source: Front Microbiol. 2016 Apr 13;7:500. doi: 10.3389/fmicb.2016.00500 (PMC4830129; doi:10.3389/fmicb.2016.00500)
Supplement: Supplementary file 1 [file Presentation_1.PDF]

Supplementary Material

Rapid Microbiome changes in freshly deposited bovine feces under field conditions

Kelvin Wong[1,2], Timothy I. Shaw[3,4], Adelumola Oladeinde[5,6], Travis C. Glenn[6], Brian Oakley[7], and Marirosa Molina[1*]

[1] United States Environmental Protection Agency, Ecosystems Research Division, 960 College Station Road, Athens, GA 30605

[2] Oak Ridge Institute for Science and Education, Oak Ridge, TN 37830

[3] Institute of Bioinformatics, University of Georgia, Athens, GA 30605

[4] Department of Computational Biology, St Jude Children's Research Hospital, Memphis, TN 38105

[5] Student Services Contractor United States Environmental Protection Agency, Ecosystems Research Division, 960 College Station Road, Athens, GA 30605

[6] Department of Environmental Health Science, Environmental Health Science Building, University of Georgia, Athens, GA 30602

[7] Western University of Health Sciences, College of Veterinary Medicine, Pomona, CA 91766

[*]Corresponding Author:

Email: molina.marirosa@epa.gov Phone: 1-706-355-8113, Fax: 1- 706-355-8104

Keywords: Metagenomics, cattle feces, community structure shift, oxygen exposure.

Running Title: Fecal microbiome under field conditions

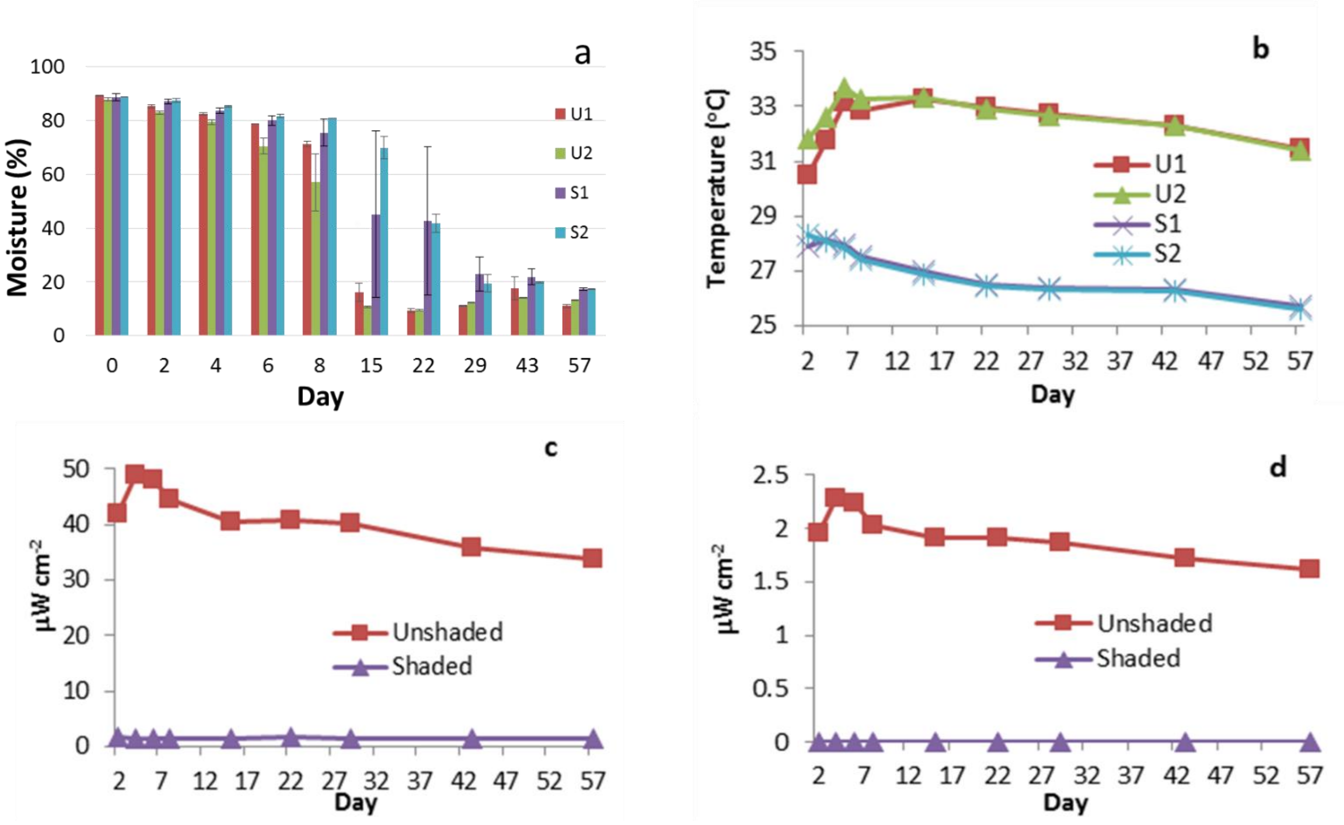

Supplementary Figure 1. UV exposure and environmental factors measured in cowpats. Moisture (a) and temperature (b) of cowpats and intensity of UVA (c) and UVB (d) at shaded and unshaded plots. Vertical bars in (a) indicate the range of moisture values from duplicate cowpats.

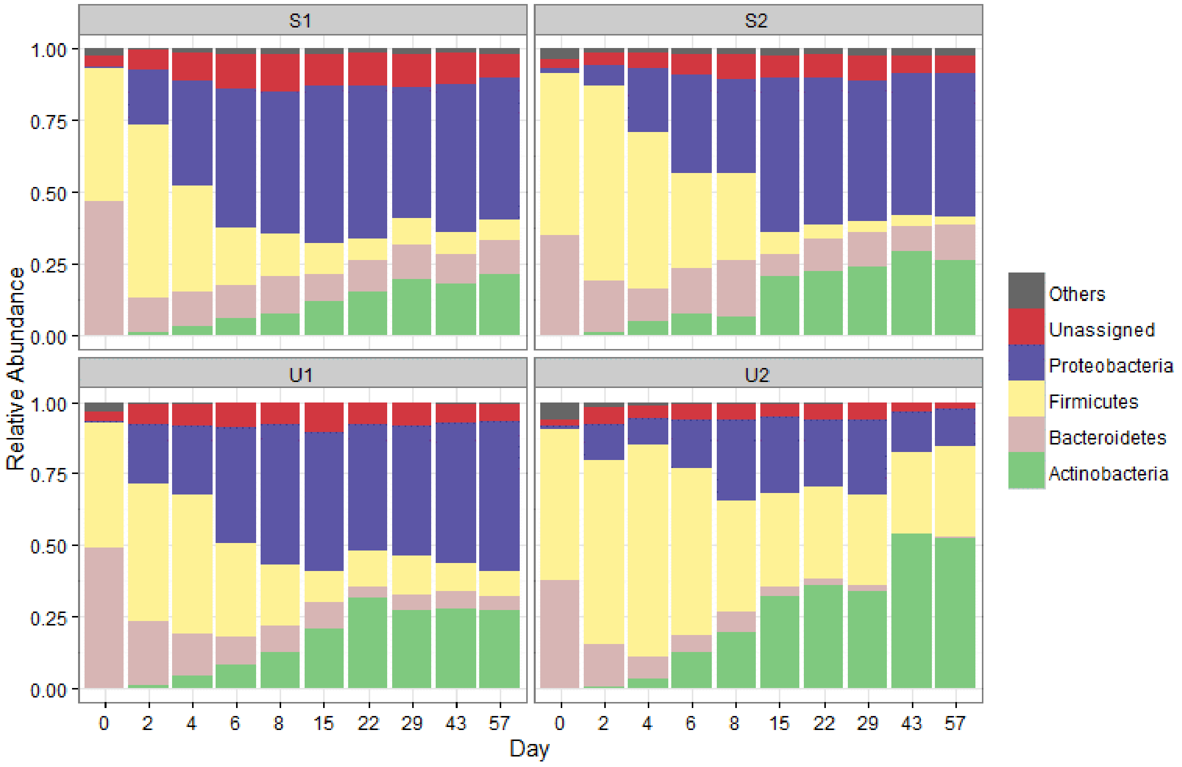

Supplementary Figure 2. Relative abundance of bacterial phylum. Relative abundance (RA) of bacterial phylum in shaded (S) and unshaded (U) samples from farms 1 and 2. "Others" are phyla with less than 0.5% RA.

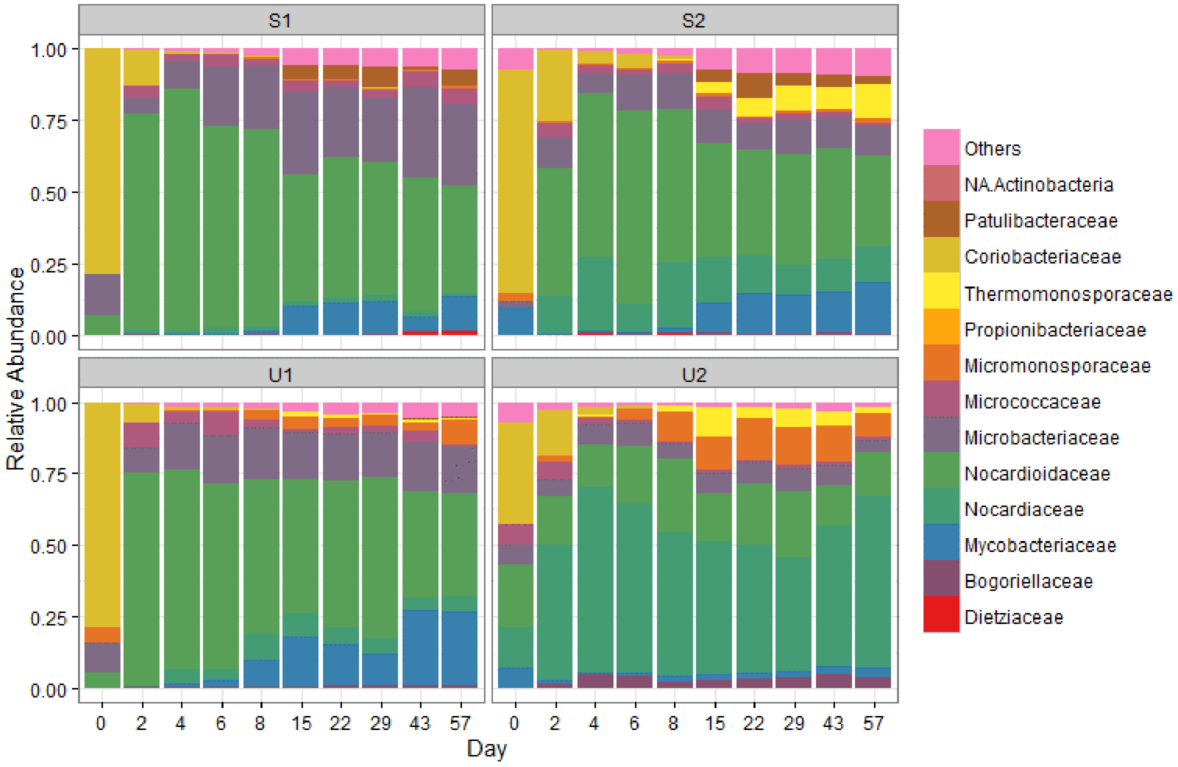

Supplementary Figure 3. Relative abundance of *Actinobacteria* families. Relative abundance (RA) of *Actinobacteria* families in shaded (S) and unshaded (U) samples from farm 1 and 2. "Others" includes all families with less than 0.5% RA. NA = not assigned.

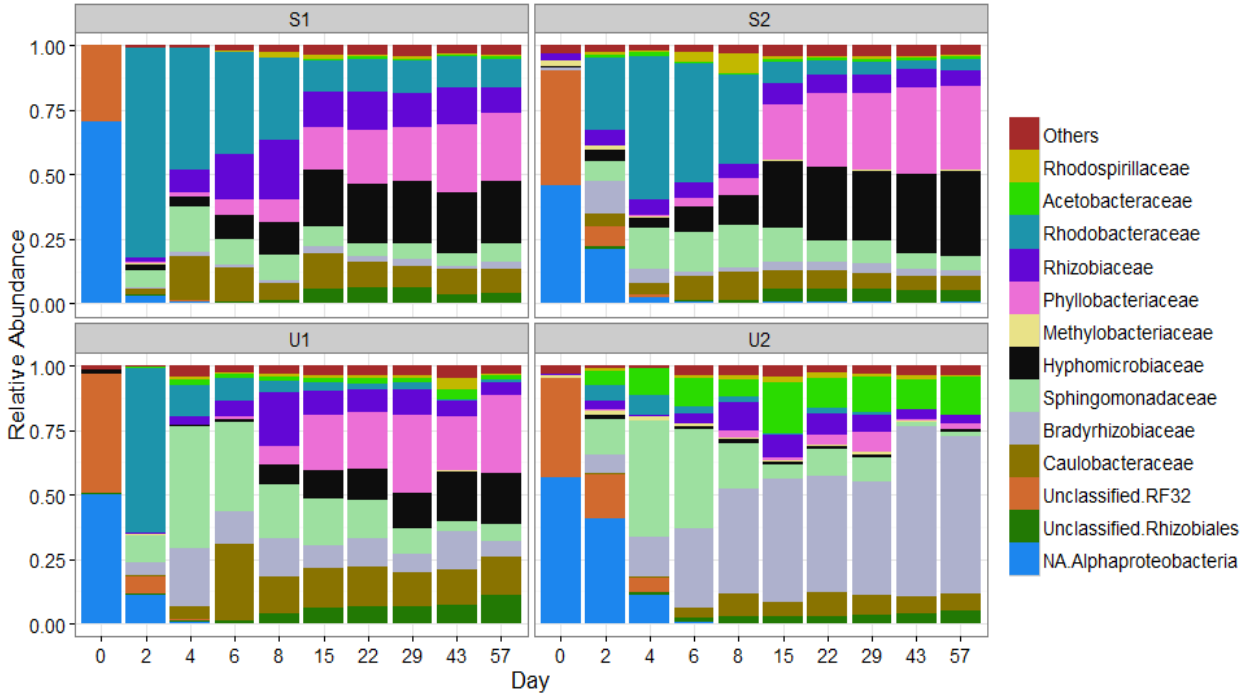

Supplementary Figure 4. Relative abundance of *Alphaproteobacteria* families. Relative abundance (RA) of *Alphaproteobacteria* families in shaded (S) and unshaded (U) samples from farm 1 and 2. “Others” includes all families with less than 0.5% RA. NA = not assigned.

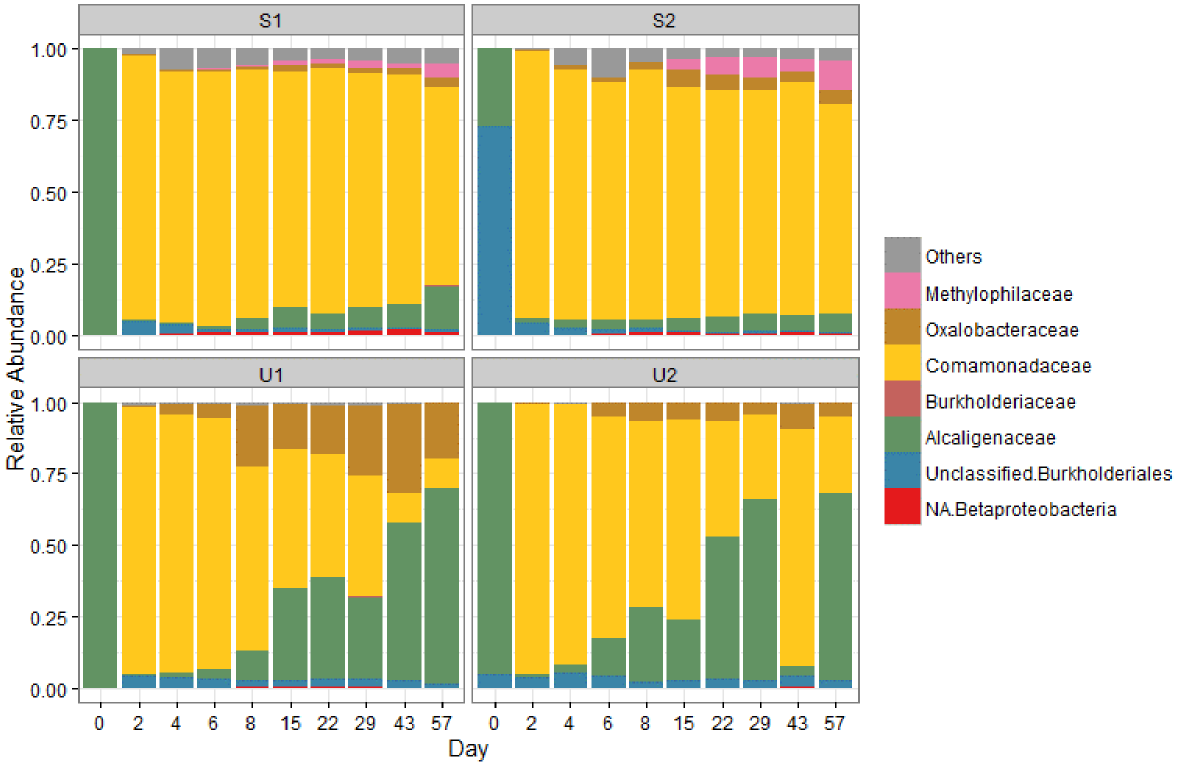

Supplementary Figure 5. Relative abundance of *Betaproteobacteria* families. Relative abundance (RA) of *Betaproteobacteria* families within shaded (S) and unshaded (U) samples from farms 1 and 2. "Others" are families with less than 0.5% RA. NA = not assigned.

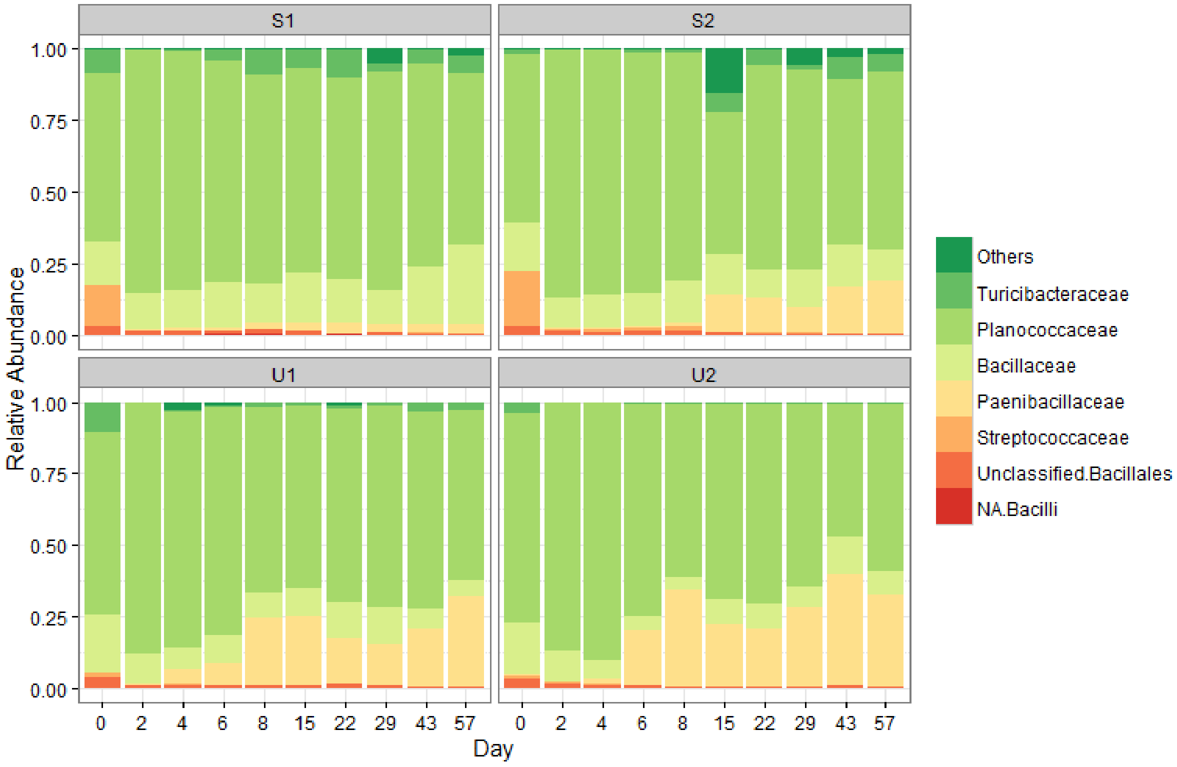

Supplementary Figure 6. Relative abundance of *Bacilli* families. Relative abundance (RA) of *Bacilli* families within shaded (S) and unshaded (U) samples from farms 1 and 2 at *Bacilli*. "Others" are families with less than 0.5% RA. NA = not assigned.

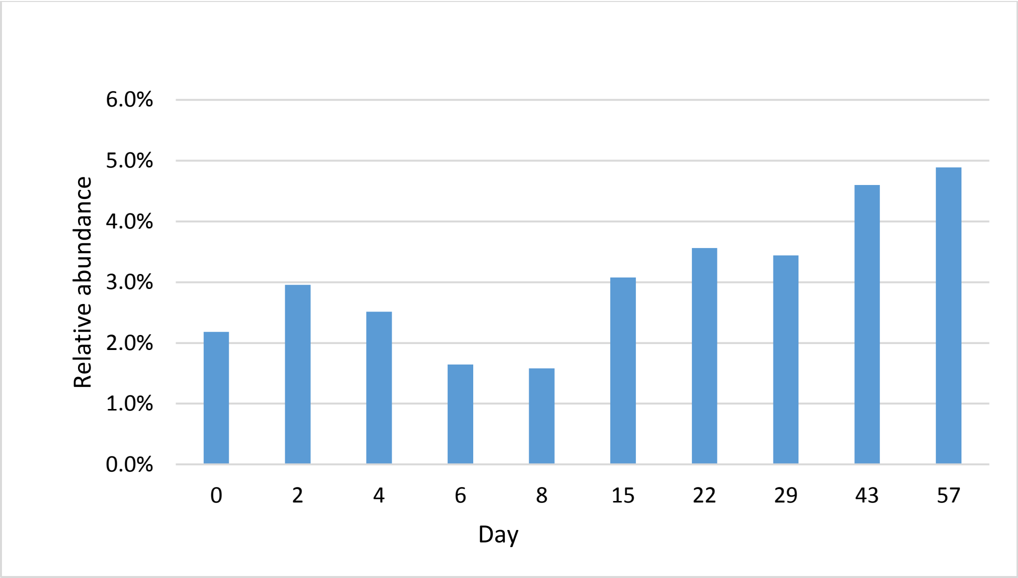

Supplementary Figure 7. Abundance of all genera consisting of pathogenic species.

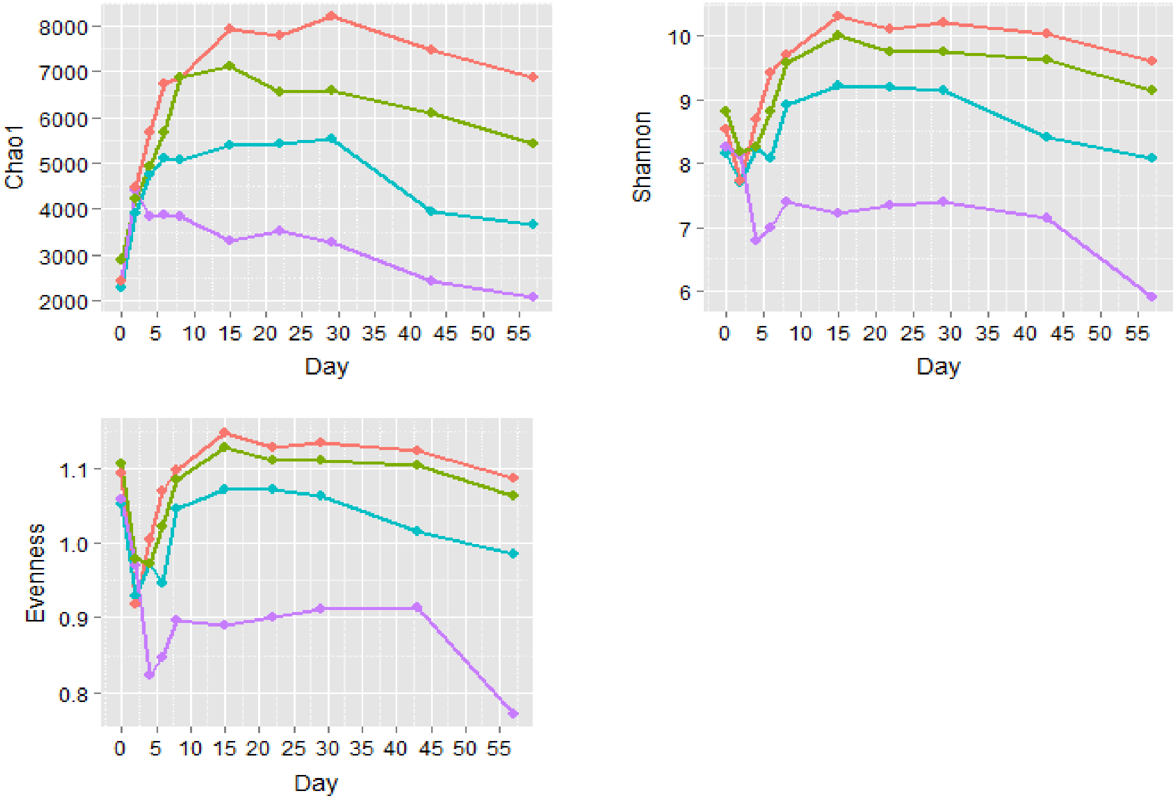

Supplementary Figure 8. Chao1, Shannon index and evenness. Changes in community diversity measured with Chao1 (Richness), Shannon index (Diversity) and evenness. Blue = unshaded farm 1 (U1), Purple = unshaded farm 2 (U2), Green = shaded farm 1 (S1), Red= shaded farm 2 (S2).

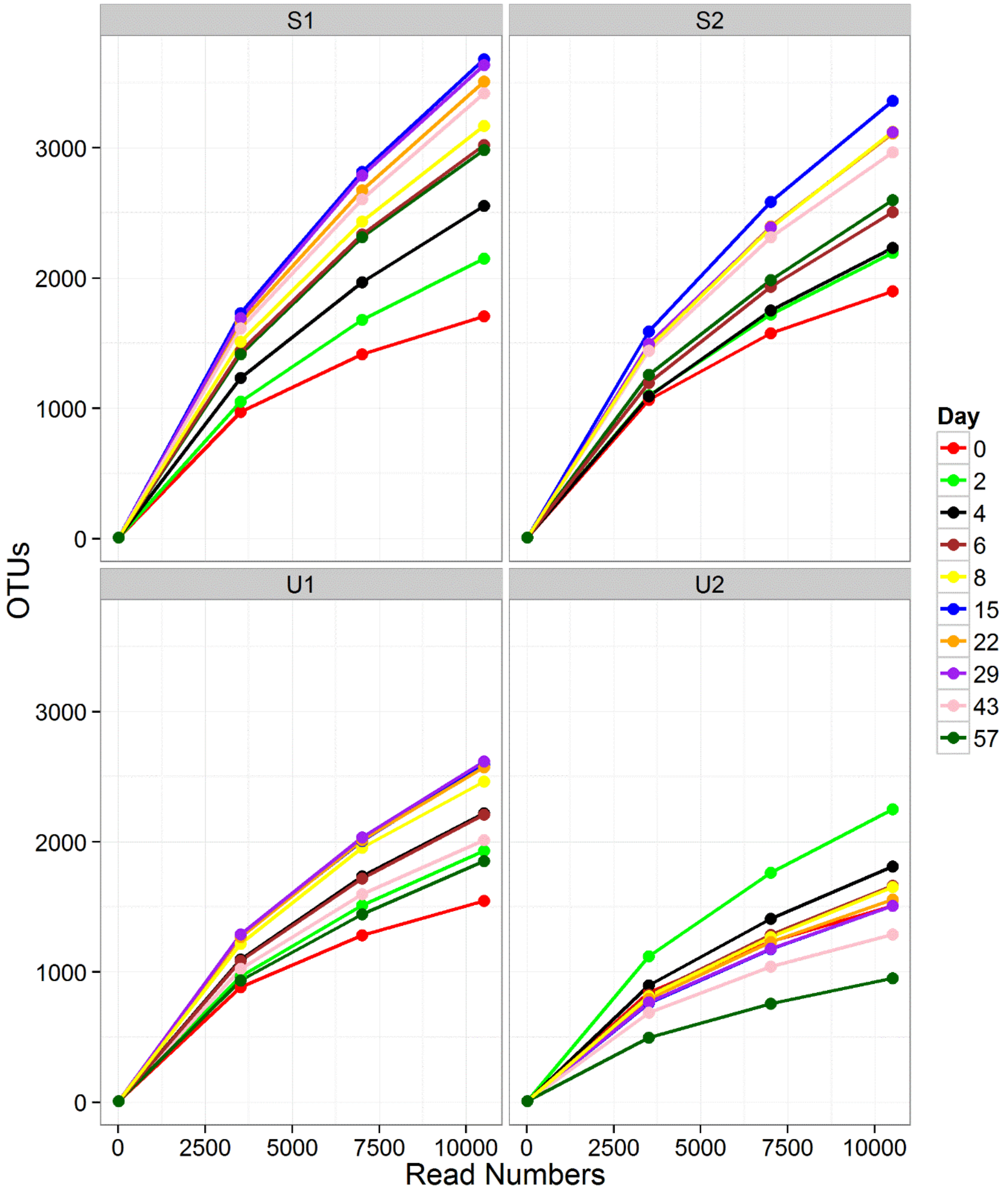

Supplementary Figure 9. Rarefaction curves at cut off levels of 3%. Rarefaction curves of shaded (S) and unshaded (U) samples from farms 1 and 2 at cut off levels of 3%.

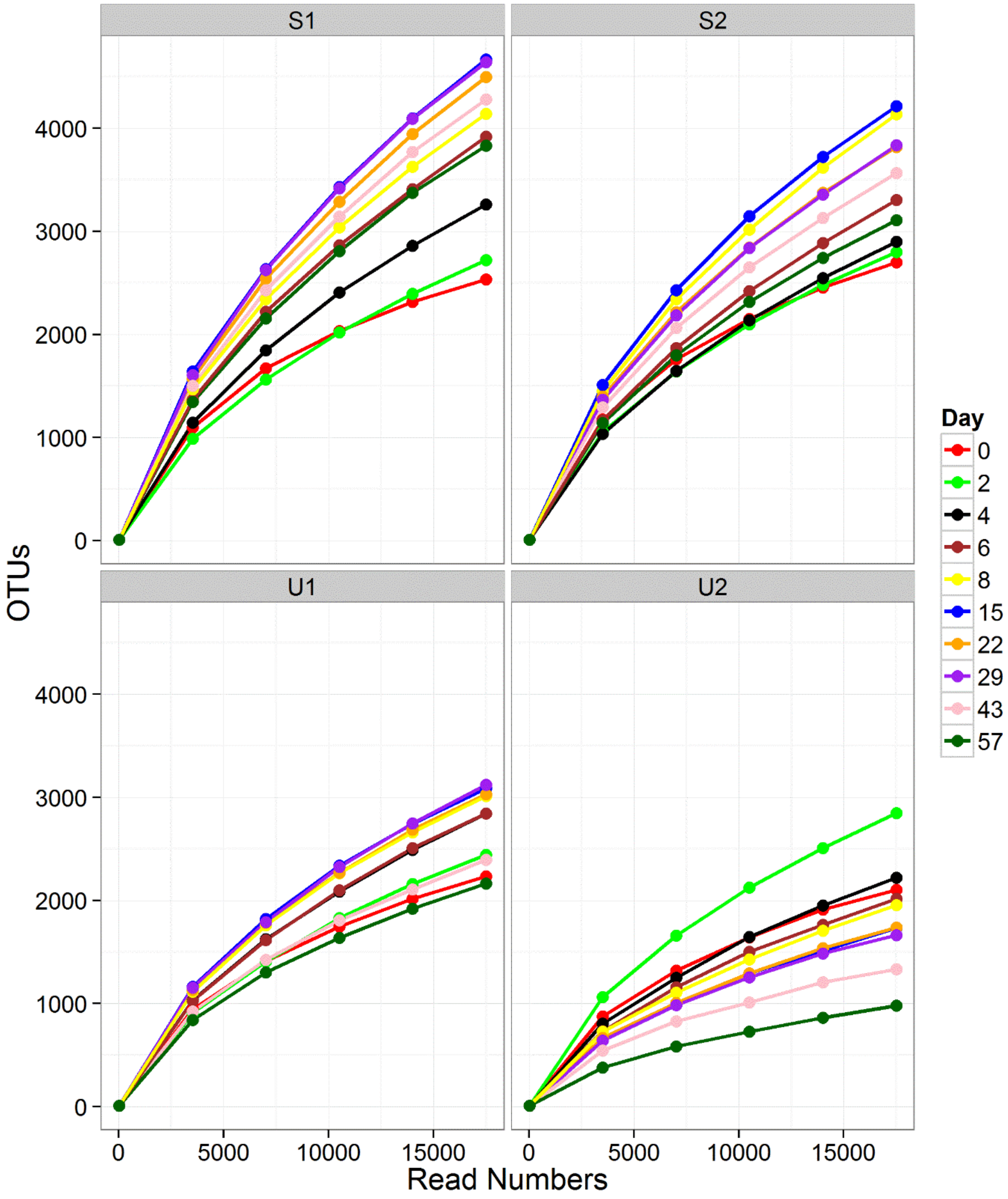

Supplementary Figure 10. Rarefaction curves at cut off levels of 5%. Rarefaction curves of shaded (S) and unshaded (U) samples from farms 1 and 2 at cut off levels of 5%.

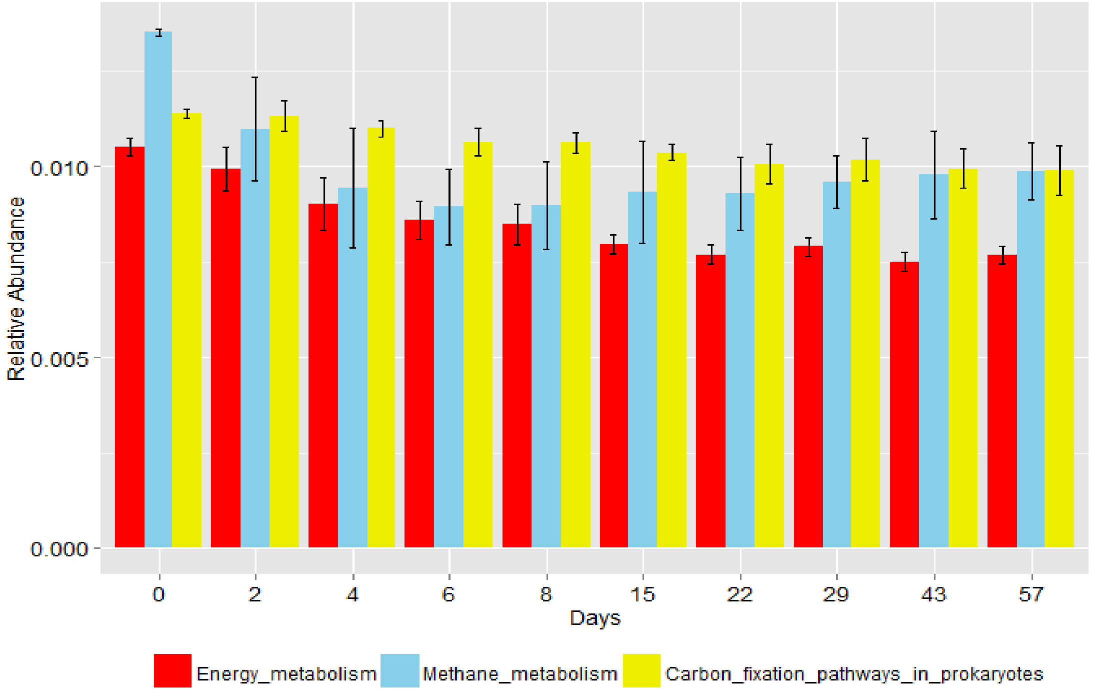

Supplementary Figure 11. Relative abundance of energy, methane and carbon fixation pathways in prokaryotes. Relative abundance (RA) of energy, methane and carbon fixation pathways in prokaryotes with shaded and unshaded samples combined (n = 8). Error bars represent the standard deviations.

Supplementary Table 1. Weight of cowpats.

Farm	Treatment	(kg)
1	Unshaded#1	1.596
	Unshaded#2	1.544
	Shaded#1	1.436
	Shaded#2	0.703
2	Unshaded#1	1.251
	Unshaded#2	1.087
	Shaded#1	1.188
	Shaded#2	1.346

Supplementary Table 2. Tag sequences for Nextera and iNEXT primers.

Nextera 1	Nextera 2	iNEXT 5	iNEXT 7
GGTAC	AGGAA	ACACGGTT	ACGAATCC
CAACAC	GAGTGG	GTCACTGT	AATGGTCG
ATCGGTT	CCACGTC	ACTGCTAG	CGCTACAT
TCGGTCAA	TTCTCAGC	GAAGAGGT	CCTAAGTC
AAGCG	CTAGG	CGCTAGTA	TTGCTTGG
GCCACA	TGCTTA	GCTGTTGT	CCTGTCAA
CTGGATG	GCGAAGT	ACATTGCG	AGCCTATC
TGATTGAC	AATCCTAT	AAGCACTG	TGATCACG
	ATCTG	CTCCTAGA	TATGGCAC
	GAGACT		ATAACGCC
	CGATTCC		GTAGTACC
	TCTCAATC		CGCGTATT
			ATCCACGA
			TAACGTCG
			CCTTCCAT
			GATCAAGG
